# The Test for Glanders by Inoculation

**Published:** 1882-10

**Authors:** 


THE TEST FOR GLANDERS BY INOCULATION.—The question, how can the veterinarian say positively whether or not a horse has the glanders, is, according to M. Ad. Rene, now settled. The dog, the friend of man, here becomes the friend of the veterinarian, and helps him out in the matter in question.

At the Veterinary School of Belgium, a series of experiments have been made to test the question whether glanders could be inoculated in the dog. Fifteen animals were used, and in each case the disease was produced.

The method of inoculation is, says Rene, of no great importance. It is enough that some of the secretion from the diseased parts be gotten in the subcutaneous tissue of the dog. The most convenient way, however, is to take a thread soaked in the glanderous secretion, and use it as a seton.

The symptoms resulting from inoculation are as follows: For two or three days there is no sign of disturbance. Then signs of fever appear, the place of inoculation swells, fluctuation is felt, a bloody and purulent fluid exudes, the tissues break down, a spreading ulcer is formed. Other ulcers develop in various parts of the body.

An important symptom is lameness, which is produced by a swelling and inflammation of one or more joints.

A catarrh of the nose may appear, but this is not invariable.

The eyes become reddened, suffused, perhaps inflamed. Ulcers of the cornea sometimes develop. The appetite is lessened, or is lost. Emaciation progresses rapidly; a fetid diarrhœa sets in.

The duration of the disease varies between one and five or even seven weeks.

Glanders is not invariably fatal in dogs. Five out of twelve recovered, in the experience of Dr. Rene.

Others have found that glanders runs a much milder course than is above described.

The post-mortem lesions are as follows: The skin shows many ulcers and farcy buds. The lungs sometimes contain tubercles; they may also show the glanderous pneumonia found in horses.

Digestive apparatus: The liver may have tubercles. There is likely to be congestion and inflammation of the lower parts of the intestine.

The lymphatic glands are enlarged and inflamed.

Many of the joints show a suppurative arthritis.

M. Rene claims that in the inoculation of the dog we have a sure test of the presence of glanders. Veterinarians should not forget, however, that some experimenters have not been able always to inoculate the disease in this animal, until there is more corroborative experience, therefore, we must not infer too surely that if the dog does not take the disease the horse does not have it.

Glanders, it is now asserted, can be transmitted to the following animals: Sheep (Bouley and Renault), goats (Gerlach and others), spontaneously in goats (Ercolani), bats (Christol and Kisner), lions and bears (Leisering, Lafosse, and others), mice, rabbits, Guinea pigs.

Attempts to inoculate cattle, swine, and domestic fowls have almost invariably failed.

